# Increasing access to screening for blood-borne viruses and sexually transmissible infections for Aboriginal and Torres Strait Islander Australians: evaluation of the Deadly Liver Mob program’s ‘cascade of care’ across nine sites in New South Wales, Australia

**DOI:** 10.1186/s12954-023-00850-6

**Published:** 2023-09-05

**Authors:** Elena Cama, Kim Beadman, Mitch Beadman, Kerri-Anne Smith, Jade Christian, Aunty Clair Jackson, Beverley Tyson, Clayton Anderson, Larissa Smyth, Jennifer Heslop, Gary Gahan, Victor Tawil, Felicity Sheaves, Louise Maher, Julie Page, Donna Tilley, Ann Ryan, Kim Grant, Basil Donovan, Annabelle Stevens, Trevor Slattery, Kate Pearce, Franklin John-Leader, Andrew Walden, Jo Lenton, Margaret Crowley, Carla Treloar

**Affiliations:** 1 Centre for Social Research in Health, UNSW Sydney, Sydney, NSW 2052 Australia; 2 Needle and Syringe Program, Mount Druitt Community Health Centre, Western Sydney Local Health District, Mount Druitt, NSW 2770 Australia; 3 Needle and Syringe Program, Nepean Blue Mountains Local Health District, Penrith, NSW 2747 Australia; 4 Dubbo Sexual Health, Western NSW Local Health District, Dubbo, NSW 2830 Australia; 5 Byron Central Hospital, Mid North Coast and Northern NSW Local Health District, Byron Bay, NSW 2481 Australia; 6 HIV & Related Programs, Mid North Coast and Northern NSW Local Health District, Coffs Harbour, NSW 2450 Australia; 7 Kirketon Road Centre, South Eastern Sydney Local Health District, Sydney, NSW 1340 Australia; 8 Centre for Population Health, Ministry of Health, Sydney, NSW 2065 Australia; 9 Western Sydney Sexual Health Centre, Western Sydney Local Health District, Sydney, NSW 2150 Australia; 10 HIV & Related Programs Unit, Western and Far West NSW Local Health District, Dubbo, NSW 2830 Australia; 11 Kirby Institute, UNSW Sydney, Sydney, NSW 2052 Australia; 12 Needle and Syringe Program, Western NSW Local Health District, Dubbo, NSW 2830 Australia; 13 Broken Hill Community Centre, Far West Local Health District, Broken Hill, NSW 2880 Australia

**Keywords:** Aboriginal and Torres Strait Islander people, Blood-borne viruses, Health promotion, Hepatitis C, Sexually transmissible infections

## Abstract

**Background:**

Aboriginal and Torres Strait Islander Australians are disproportionately impacted by blood-borne viruses (BBVs) and sexually transmissible infections (STIs). Stigma remains one of the key barriers to testing and treatment for BBVs and STIs, particularly among Aboriginal and Torres Strait Islander people. The Deadly Liver Mob (DLM) is a peer-delivered incentivised health promotion program by and for Aboriginal and Torres Strait Islander Australians. The program aims to increase access to BBV and STI education, screening, treatment, and vaccination for Aboriginal and Torres Strait Islander Australians in recognition of the systemic barriers for First Nations people to primary care, including BBV- and STI-related stigma, and institutional racism. This paper presents routinely collected data across nine sites on the ‘cascade of care’ progression of Aboriginal and Torres Strait Islander clients through the DLM program: hepatitis C education, screening, returning for results, and recruitment of peers.

**Methods:**

Routinely collected data were collated from each of the DLM sites, including date of attendance, basic demographic characteristics, eligibility for the program, recruitment of others, and engagement in the cascade of care.

**Results:**

Between 2013 and 2020, a total of 1787 Aboriginal and Torres Strait Islander clients were educated as part of DLM, of which 74% went on to be screened and 42% (or 57% of those screened) returned to receive their results. The total monetary investment of the cascade of care progression was approximately $56,220. Data highlight the positive impacts of the DLM program for engagement in screening, highlighting the need for culturally sensitive, and safe programs led by and for Aboriginal and Torres Strait Islander people. However, the data also indicate the points at which clients ‘fall off’ the cascade, underscoring the need to address any remaining barriers to care.

**Conclusions:**

The DLM program shows promise in acting as a ‘one stop shop’ in addressing the needs of Aboriginal and Torres Strait Islander people in relation to BBVs and STIs. Future implementation could focus on addressing any potential barriers to participation in the program, such as co-location of services and transportation.

## Introduction

Although representing 3.2% of the Australian population [1], Aboriginal and Torres Strait Islander Australians are disproportionately impacted by blood-borne viruses (BBVs) and sexually transmissible infections (STIs). Notification rates of chlamydia, gonorrhea, syphilis, hepatitis B virus (HBV), hepatitis C virus (HCV), and HIV^1^ among Aboriginal and Torres Strait Islander people are diagnosed at 2.8, 4.2, 5.5, 1.8, 5.9, and 1.6 times, respectively, the rates of non-Aboriginal and Torres Strait Islander people in Australia [2, 3]. Rates of HCV have been found to consistently higher among Aboriginal and Torres Strait Islander people who inject drugs between 2018 and 2022 (range 36–53%) compared to non-Aboriginal or Torres Strait Islander people who inject drugs (range 31–44%) [4]. Although lifetime testing for HCV is comparable, recent RNA testing is suboptimal, and treatment uptake has been found to be substantially lower among Aboriginal and Torres Strait Islander people living with chronic HCV compared to non-Aboriginal or Torres Strait Islander people [5]. As a result of high prevalence and incidence rates, and lower treatment uptake, Aboriginal and Torres Strait Islander Australians remain a priority population for the national and New South Wales (NSW) strategies for addressing BBVs and STIs [6, 7].

However, racism and BBV- and STI-related stigma can act as significant barriers to First Nations people seeking testing and treatment [8–10]. First Nations people may experience multiple and intersecting forms of stigma and marginalisation, which is further exacerbated by ongoing institutional racism, which refers to the ways that our institutions are explicitly and implicitly underpinned by racist beliefs and values [11]. Beyond racism and stigma, First Nations communities may face additional barriers to accessing mainstream health services, including lack of culturally sensitive and safe resources and care, costs and affordability, distance and lack of transportation, and mistrust in non-First Nations health workers [12–14]. It is apparent that to address these health issues, health systems must first address ongoing BBV- and STI-related stigma and institutional racism, by providing culturally sensitive and safe opportunities for Aboriginal and Torres Strait Islander people to engage in health care.

To provide a culturally sensitive and safe space for Aboriginal and Torres Strait Islander people to engage in care for BBVs and STIs, the Deadly Liver Mob (DLM) program was first introduced as a pilot program in 2013 in one publicly funded needle and syringe program (NSP). DLM program is a peer-led health promotion program that aims to increase access to screening, treatment, and vaccination for BBVs and STIs for Aboriginal and Torres Strait Islander people through mainstream NSP and sexual health services. The program was initially modeled from the Safe Injecting Cwiz (SIC) conducted in western Sydney between 1998 and 2002, which targeted people under the age of 25 years who injected drugs [15]. The SIC was, in turn, adapted from a US-based HIV peer-driven intervention for people who inject drugs, which attempted to reach hidden networks [16–18].

In the DLM program, Aboriginal and Torres Strait Islander clients who meet eligibility criteria are invited to a HCV education session with an Aboriginal and Torres Strait Islander worker and encouraged to refer their peers to the program. The presence of Aboriginal and Torres Strait Islander workers allows for culturally safe and appropriate introductions of clients to the health service [19]. Although the focus of DLM is largely on HCV, after the education session, clients are offered referral to sexual health services for BBV and STI assessment and screening. Sexual health staff then manages screening, return of results, provision of treatment for STIs (if required), and provision of hepatitis A virus (HAV) or HBV vaccination. Staff makes attempts to follow-up clients to return for screening, results, and follow-up care. Clients are offered nominal incentives at each stage of the program; education, recruitment of peers, screening, treatment, and vaccination (see [19] for program model).

The DLM program began as two pilot sites within western Sydney, NSW, in 2013 and 2015, respectively. In the first 12 months at the first pilot sites, more than 400 Aboriginal and Torres Strait Islander people received education, and over 300 people were referred to sexual health screening, resulting in a 1023% increase in access to sexual health services. Findings from an early mixed methods evaluation of the two pilot sites showed that the program was well-received by Aboriginal and Torres Strait Islander clients and resulted in increased attendance and engagement within the service [19]. As part of a National Health and Medical Research Council (NHMRC) Partnership Grant, funding enabled expansion of the program to run in nine sites within seven Local Health Districts (LHDs)^2^ in NSW who were partners on the grant: three in Sydney metropolitan area and six in rural and regional NSW. The program is based in NSPs, sexual health clinics, community health centers, and through outreach. This paper uses data collected as part of an evaluation of the implementation of DLM across the nine sites in relation to clients’ attendance in the various stages of the DLM program [20]. This paper evaluates clients’ ‘cascade of care’ progression (i.e., engaging in hepatitis C education, screening, returning for results, peer referral) and monetary investment required using routinely collected health service data from the DLM sites.

## Methods

The broader Partnership Project, led by the Centre for Social Research in Health, sought to evaluate the health outcomes and impacts of DLM, examine its acceptability, and develop a scale up and implementation toolkit that could be used at future DLM sites. This quantitative component includes analyses of routinely collected data by each of the DLM sites and shows clients’ engagement in the DLM program, including engagement in education, screening, and returning for results, and peer referral.

### Procedure and materials

Aboriginal and Torres Strait Islander people who met eligibility criteria for the program were invited to a hepatitis C education session with a DLM Aboriginal and Torres Strait Islander health worker. Aboriginality is determined by the local Aboriginal DLM workers and in accordance with community principles. Due to the focus of the program on HCV screening and treatment, Aboriginal and Torres Strait Islander clients must:

1. Have ever injected drugs; OR
2. Currently inject drugs; OR
3. Are classified as ‘at risk’ of injecting drug use and/or BBVs and/or STIs. ‘At risk’ was defined as having ever been in prison, having had an unsafe tattoo, or living with a person/people who inject drugs and/or has HCV. ‘At risk’ was a working definition, with the possibility of evolving based on any new knowledge.

Non-Aboriginal or Torres Strait Islander romantic and/or sexual partners of Aboriginal and Torres Strait Islander clients are permitted to take part in the DLM program due to the importance of BBV and STI screening of sexual partners [21–23]. However, non-Aboriginal or Torres Strait Islander people are not provided with incentives to participate, and data on non-Aboriginal or Torres Strait Islander people were not recorded as part of the evaluation.

The DLM program is designed to be a low-threshold service, with minimal client data collected in order to remove barriers to engagement in primary care. The evaluation attempts to mirror this approach with the research design, so as to ensure the research was not adding any barriers to clients engaging in the program. Routinely collected de-identified data were provided by each site to the evaluation team, rather than obtaining individual consent from clients to access these data. A waiver of consent was approved by the ethics committees overseeing the project. Ethics approval for the evaluation of the DLM program was obtained from South Eastern Sydney LHD and the Aboriginal Health and Medical Research Council Ethics Committees. Site-specific approvals were also obtained from each of the seven LHDs governing the nine sites involved in the study.

Routinely collected data include the date the client attended the program, basic demographic characteristics (age, sex, and postcode of residence), eligibility for the program or risk markers for injecting drug use or BBVs and STIs (defined above), recruitment of others to the program (maximum of three people recruited), and their engagement in the DLM program (e.g., education, screening, returning for results, receiving follow-up sexual health, and incentive payments provided for participation). Follow-up sexual health could include vaccination for HAV and HBV, and treatment for STIs if required (e.g., prescriptions for medication). The provision of HCV treatment has changed significantly since the introduction of direct-acting antiviral therapy (DAAs), with the Australian Government subsidising treatment without restrictions from 2016. DAAs can now be prescribed in non-specialist and primary care settings including NSPs, sexual health, and by general practitioners [24]. This has increased access to and uptake of treatment for HCV (see [25] for latest treatment numbers), but has made tracking of DLM clients through to HCV treatment challenging. Incentive vouchers are thus not provided for HCV treatment. Two sites commenced the program prior to the introduction of DAAs for HCV, and these sites offered refresher education to any returning clients to provide updated treatment information. Although these sites provide incentive payments to clients returning for refresher education, these payments are not recorded in this paper given that they are not considered standard practice as part of DLM. This paper reports on incentive payments provided to clients for engagement in the program, but it should be noted that program costs also include staffing and laboratory testing, which are not included in this paper.

Data from the introduction of DLM as a pilot site on April 29, 2013, up to June 30, 2020, where all nine sites were running are included in this paper. The program start dates for the nine sites were (1) April 29, 2013 (metro area); (2) February 12, 2015 (metro area); (3) and (4) February 8, 2017 (regional area); (5) February 15, 2017 (regional area); (6) and (7) September 11, 2017 (regional area); (8) September 19, 2017 (regional/rural area); and (9) February 27, 2018 (metro area).

### Data analysis

Routine data recorded by the sites were originally collected on paper-based DLM intake forms and then entered into Microsoft Excel spreadsheets by DLM staff. The intake forms and spreadsheets were somewhat standardised across all the sites due to the information sharing that occurred at the beginning of the Partnership Project; however, there were some variations based on local requirements or preferences. The earliest pilot site which began in 2013 had incomplete demographic data due to differences in data recording at the beginning of program implementation. The spreadsheets were de-identified by DLM staff (all identifying client information was removed) and password protected, before being transferred to the evaluation research staff.

The data were examined in relation to the notion of the ‘treatment cascade’ or ‘cascade of care’ [26]. The idea of cascade is to draw attention to the multiple points at which a person may ‘fall off’ the care and treatment pathway. Interview data with DLM staff and clients are explored elsewhere [20]. The ‘cascade of care’ progression refers to clients’ engaging in education, BBV/STI screening, and returning for their results. Not all sites collect data on additional treatment required by DLM clients, such as STI treatment and HAV or HBV vaccination. This is due to the anonymous nature of some sexual health services. Further, not all clients will require additional treatment. Thus, data on additional treatment are not presented. The data presented include demographic and risk characteristics as well as engagement of clients in the various components of DLM, including frequency, percentage, and incentives provided for each stage (excluding additional treatment as noted above) as well as for peer referral. Valid percentages are reported, to account for some missing data. Graphs present data across all nine sites situated within seven LHDs, and sites are not individually identified. Some of the data is only presented since 2016 when the sites standardised data collection across all DLM sites, and this is noted where applicable.

Comparisons in engagement in screening and returning for results between men and women were conducted using Chi-square analyses. Point biserial correlation was used to examine the relationship between age and engagement in screening and returning for results. These analyses were not conducted for engagement in education as all clients who enter DLM receive education. Analyses were conducted by the first author (EC), then workshopped with Aboriginal and Torres Strait Islander authors (KB and MB) and non-Aboriginal or Torres Strait Islander authors (CT and EC).

## Results

### Demographic and risk characteristics of DLM clients

Demographic and risk factor data for a subset of DLM clients who entered the program since the introduction of the NHMRC Partnership Project (*n* = 985) in 2016 up to the end of data collection in 2020 are presented in Table 1. This is the timepoint at which data collection was standardised across all the sites. Prior to this time, demographic and risk data were not necessarily routinely collected.

**Table 1:** Demographic characteristics of DLM clients

*N* = 985	*N* (%)
Age M (SD), range	37.17 (11.20), 14–76
Gender	
Male	494 (50)
Female	488 (50)
Current or previous injecting drug use	630 (70)
Prison history	416 (49)
Unsafe tattoo	427 (49)
Living with person who injects or who has HCV	331 (37)

Valid percentages are reported. Gender was collected as a binary variable, and thus, gender diversity was not recorded

### Cascade of care progression

**Fig. 1:**
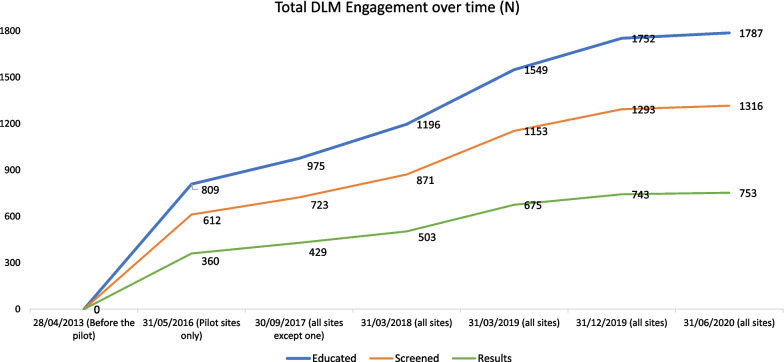
Cumulative total DLM engagement across the four data reports

Figure 1 shows the total DLM engagement across the life of the project, marked by six timepoints. The timespan is from April 29, 2013 (when the first pilot site commenced), to July 30, 2020, when the final data capture took place (seven sites represented). The figure shows the cumulative numbers of clients who have been educated, screened for BBVs and STIs, and who returned to receive the results of their screening. The six key timepoints are as follows:

1. Prior to pilot commencement: to show no DLM intervention
2. Two pilot sites operating
3. Eight sites operating
4. Nine sites operating
5. Nine sites operating
6. Final data capture: nine sites operating

Data show that a total of 1787 Aboriginal and Torres Strait Islander clients entered the program and received HCV education across all nine sites between 2013 and 2020. Of the total number of clients, 1316 received BBV and STI screening, and 753 returned to receive their results. It is important to note that the coronavirus pandemic disrupted provision of DLM from March 2020 onwards, with many sites ceasing operations, particularly during stay-at-home and lockdown orders during the pandemic. While some sites have recommenced DLM operations, others have absorbed learnings of DLM into other programs instead.

**Fig. 2:**
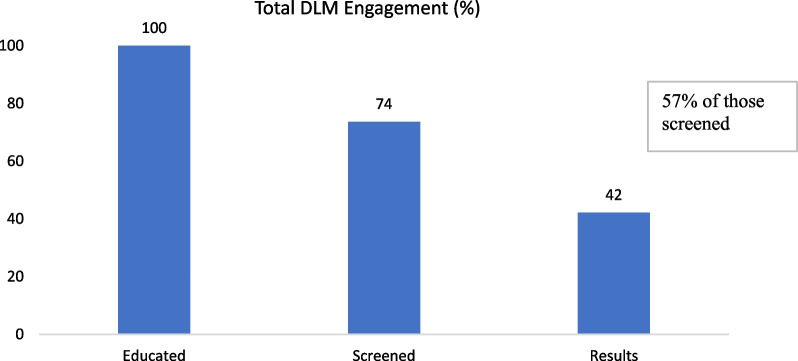
‘Cascade of care’: proportion of DLM clients attending each step of the program, all sites

Figure 2 shows the ‘cascade of care’ progression of DLM clients. Of the 1787 Aboriginal and Torres Strait Islander clients who were educated, 74% went on to be screened, and 42% of the total (or 57% of those screened) returned to receive their results.

There is significant variation in proportions of people who go on to receiving screening and return for their results across the sites. The proportion of clients who went on to be screened ranges from 40% to 87% across the sites, while the proportion of those who return for their results ranges from 15% to 66% of total clients (or 36% to 79% of those screened). Although DLM staff made attempts to engage DLM clients to return for screening and results, these efforts were not always successful. We describe some of the individual and systemic barriers to engagement in DLM elsewhere, such as geographic spread of clients (i.e., travel distance to sites), travel cost, and parking difficulties, among others [20]. Sites made efforts to address some of these challenges, such as by doing outreach to more regional and remote areas.

We examined differences in engagement in screening and results according to age and gender. Given these data were only routinely collected after 2016, these analyses were limited to clients who entered the program between 2016 and 2020 (*n* = 985). There were no differences in men and women’s engagement in screening or returning for results (*p* > 0.05). There was also no association between age and engagement in screening and returning for results (*p* > 0.05).

### Incentive payments for cascade of care

Over the life span over the project (from 2013 and 2020), an estimated $56,220 was spent in incentive payments to Aboriginal and Torres Strait Islander clients in the DLM program for engagement in the cascade of care. Of this amount, $35,640 was spent on incentive payments for HCV education of clients, $13,100 was spent on incentives for screening, and $7480 was spend on incentives for clients returning to receive their results.

### Incentive payments for peer recruitment

Clients have the opportunity to refer up to three peers to the program for an additional $10 per recruit. For peer referral data, we focus on recruitment by any clients who entered the program since the introduction of the NHMRC Partnership Project (*n* = 985) in 2016. This was because early DLM sites allowed additional recruitment beyond three clients, and this was standardised in 2016 across all sites for up to three recruits per client. Since 2016, a total of 149 clients (15%) referred others to the program: of these, 106 (71%) recruited one person, 29 (20%) recruited two people, and 14 (9%) recruited three people. This comes to a total of $1596 in peer recruitment costs.

We also include prospective costs here to illustrate the potential variability in these costs depending on how many people others recruit to the program. For instance, out of the 985 clients who entered the program since 2016, they could have together recruited anywhere between 985 and 2955 others at a total cost between $9850 and $29,550. Out of the 1787 clients who entered the program across the life of the project (from 2013 to 2020), they could have recruited 1787–5361 clients. This would have come to a cost ranging from $17,870 to $53,610.

## Discussion

The aim of this paper was to evaluate the ‘cascade of care’ progression of Aboriginal and Torres Strait Islander clients through the DLM program, and the estimated monetary investment required for clients’ progression through the core components of the program. Routine data collected by the sites where DLM was operating between 2013 and 2020 indicate that a total of 1787 Aboriginal and Torres Strait Islander people entered the DLM program and received HCV education, 1316 received BBV and STI screening (74%), and 753 returned to receive their results (42% of total; 57% of those screened). The DLM program aims to overcome some of the systemic barriers to health-care engagement and retention for Aboriginal and Torres Strait Islander Australians. The data presented in this paper show that providing a culturally sensitive and safe space for Aboriginal and Torres Strait Islander people to engage in BBV and STI screening and treatment has positive impacts on engagement and retention, demonstrated through the progression through the cascade of care, requiring return visits from clients.

As we have discussed elsewhere in relation to the two pilot sites [19], engagement in DLM may initially be high immediately after the program’s introduction but may stabilise or later decrease over time as saturation in the local community is reached. It is important to understand that while these numbers may decrease over time, they nevertheless remain higher than at baseline, suggesting that the service is able to engage and retain clients over time. This is also demonstrated through provision of refresher education to returning clients within the two pilot sites of the program, both of which commenced the DLM program prior to the introduction of DAAs. In light of the above, what constitutes success in implementation of the DLM program must be carefully considered. In this paper, we have presented cumulative engagement in the program to reflect this success. Future sites must establish baseline rates of attendance to adequately measure the impacts of the DLM program.

The data highlight multiple points at which people may ‘fall off’ the ‘cascade of care.’ Within some sites, the proportions of clients who go on to receive BBV and STI screening and return for their results may be lower than others as shown in the results. There may be multiple explanations for this. For example, not all DLM sites have sexual health services that are co-located to the delivery of the DLM education, which presents a barrier for clients progressing to screening. Sites typically rely on a single (or up to two) local Aboriginal or Torres Strait Islander DLM workers to deliver the program, which has been hailed as one of the key benefits in engaging community [19]. However, there were numerous service interruptions during the project, such as due to staff requiring time off for personal health or family reasons, where staff left their position and the site took time to recruit a new worker, and following the coronavirus pandemic in 2020 where sites ceased operations. Where a dedicated Aboriginal or Torres Strait Islander staff member was not available to deliver the program, there may have been reduced engagement in the service. Additionally, six sites are located in rural and regional parts of the state, with clients traveling quite far distances to access services and return for results (some results were provided by phone with the client’s permission). Some sites conduct outreach activities, where they may engage clients for education and screening but have subsequent difficulties in following clients up to provide them with their results or follow-up care. The program is designed to be flexible and adaptable to the local context. Some sites adjusted the incentive amounts, whereby clients received a lower amount for education than for screening and results, to encourage clients to be screened and return for their results; these incentives provided financial support for people to return for results, especially in regional/rural areas where travel might involve significant distance and cost. These data highlight the importance of including multiple forms of data collection, particularly qualitative interviews (which will be detailed elsewhere, [20]), in order to gain a better understanding of the barriers and facilitators to clients’ engagement in all stages of the program. In the future, new technologies, such as point of care testing for HCV, may go some ways to addressing these barriers [27, 28].

There are several study limitations that must be noted. This paper draws from routinely collected data from the DLM sites. This relies on DLM staff to consistently and accurately collect and record clients’ engagement in the program and the incentives distributed. Our experience indicates that this is not always the case. For example, the types of information initially collected by the two pilot sites differed from that collected later when new sites were introduced, and thus, we do not have complete demographic data for clients who entered the program between 2013 and 2015. Sites benefited from the funding provided by the Partnership Grant, which allowed for regular catch ups between all sites and research staff. Future DLM sites may not have access to these resources. However, to assist future DLM sites with implementation of the program, we have developed an implementation toolkit, containing a range of resources and templates to guide any potential future sites (see https://www.deadlylivermob.org). An additional limitation of this data is the inability to capture further client data on treatment of HCV. This is because of the changing landscape of HCV treatment since the introduction of DAAs from March 2016, with treatment now able to be prescribed both in specialist and non-specialist settings, including NSPs, general practice, outreach clinics, sexual health, and liver clinics. Future DLM sites should consider how such data could be accurately captured prior to implementation while operating a low-threshold approach to service access (that is, not adding additional burden or barriers to clients entering a service).

The DLM program sought to promote a culturally appropriate and welcoming service for Aboriginal and Torres Strait Islander people living with or at risk of HCV through re-design of the service, tackling stigma at multiple levels, including within the organisation and delivery of health services and via the provision of incentives, acknowledging the structural impact of material deprivation for First Nations people [29]. The data presented in this paper and elsewhere [19] suggest that the DLM program and all staff involved in its implementation and delivery have been successful in achieving this goal. Future iterations of the DLM or similar programs should focus on addressing the points at which people ‘fall off’ the cascade by attending to potential barriers to participation. This could involve ensuring co-location of services and the introduction of transport support for clients, as these were identified as key challenges for participation in the DLM program. Any new sites seeking to introduce the DLM or similar programs could also look to integrating the program into comprehensive care to address multiple health needs faced by Aboriginal and Torres Strait Islander Australians.

## Conclusion

The findings presented here illustrate the positive impacts of the DLM program on Aboriginal and Torres Strait Islander peoples’ engagement in BBV and STI education and screening within primary care. The trust established between the Aboriginal and Torres Strait Islander DLM program staff and clients has been previously shown to be critical and accounts for much of the program’s success, in addition to the program’s partnership between NSP and sexual health [19]. The program has the potential to retain clients’ engagement in mainstream health services beyond the program if this trust can be maintained. The DLM program, therefore, shows promise in its potential to act as a ‘one stop shop’ in addressing Aboriginal and Torres Strait Islander community needs in relation to BBVs and STIs.

## Data Availability

The datasets generated and/or analyzed during the current study are not publicly available due to the possibility of individual privacy being compromised.

## Abbreviations

ACCESS: Australian Collaboration for Coordinated Enhanced Sentinel Surveillance of Sexually Transmissible Infections and Blood-Borne Viruses
BBV: Blood-borne virus
DAAs: Direct-acting antiviral
DLM: Deadly Liver Mob
HAV: Hepatitis A
HBV: Hepatitis B
HCV: Hepatitis C
LHD: Local Health District
NHMRC: National Health and Medical Research Council
NSP: Needle and Syringe Program
NSW: New South Wales
SIC: Safe Injecting Cwiz
STI: Sexually transmissible infection

## Footnotes

1 This figure is based on the 2018 report as the 2021 report is based on small numbers. 2021 and 2022 reports suggest that HIV notifications are similar among Aboriginal and Torres Strait Islander and non-Aboriginal or Torres Strait Islander Australians; however, these data are based on small numbers, and the report suggests may not be reflective of national trends (Kirby Institute, 2021, 2022a).

2 LHDs manage any public hospitals and health services within defined geographical areas in NSW. There are currently 15 LHDs; eight covering Sydney metropolitan area and seven covering rural and regional NSW (see https://www.health.nsw.gov.au/lhd/Pages/default.aspx).
