# Correction: Increasing access to screening for blood-borne viruses and sexually transmissible infections for Aboriginal and Torres Strait Islander Australians: evaluation of the Deadly Liver Mob program’s ‘cascade of care’ across nine sites in New South Wales, Australia

**DOI:** 10.1186/s12954-024-00927-w

**Published:** 2024-02-07

**Authors:** Elena Cama, Kim Beadman, Mitch Beadman, Kerri-Anne Smith, Jade Christian, Aunty Clair Jackson, Beverley Tyson, Clayton Anderson, Larissa Smyth, Jennifer Heslop, Gary Gahan, Victor Tawil, Felicity Sheaves, Louise Maher, Julie Page, Donna Tilley, Ann Ryan, Kim Grant, Basil Donovan, Annabelle Stevens, Trevor Slattery, Kate Pearce, Franklin John-Leader, Andrew Walden, Jo Lenton, Margaret Crowley, Carla Treloar

**Affiliations:** 1 Centre for Social Research in Health, UNSW Sydney, Sydney, NSW 2052 Australia; 2 Needle and Syringe Program, Mount Druitt Community Health Centre, Western Sydney Local Health District, Mount Druitt, NSW 2770 Australia; 3 Needle and Syringe Program, Nepean Blue Mountains Local Health District, Penrith, NSW 2747 Australia; 4 Dubbo Sexual Health, Western NSW Local Health District, Dubbo, NSW 2830 Australia; 5 Byron Central Hospital, Mid North Coast and Northern NSW Local Health District, Byron Bay, NSW 2481 Australia; 6 HIV & Related Programs, Mid North Coast and Northern NSW Local Health District, Coffs Harbour, NSW 2450 Australia; 7 Kirketon Road Centre, South Eastern Sydney Local Health District, Sydney, NSW 1340 Australia; 8 Centre for Population Health, Ministry of Health, Sydney, NSW 2065 Australia; 9 Western Sydney Sexual Health Centre, Western Sydney Local Health District, Sydney, NSW 2150 Australia; 10 HIV & Related Programs Unit, Western and Far West NSW Local Health District, Dubbo, NSW 2830 Australia; 11 Kirby Institute, UNSW Sydney, Sydney, NSW 2052 Australia; 12 Needle and Syringe Program, Western NSW Local Health District, Dubbo, NSW 2830 Australia; 13 Broken Hill Community Centre, Far West Local Health District, Broken Hill, NSW 2880 Australia

**Correction: Harm Reduction Journal (2023) 20:125** 10.1186/s12954-023-00850-6

Following publication of the original article [1], the reference 20 has been added to the reference list and the same has been shown below:

20. Treloar, C., Beadman, K., Beadman, M. et al. Evaluating a complex health promotion program to reduce hepatitis C among Aboriginal and Torres Strait Islander peoples in New South Wales, Australia: the Deadly Liver Mob. Harm Reduct J 20, 153 (2023). 10.1186/s12954-023-00885-9

Also, affiliation for the author Annabelle Stevens has been changed from "Kirketon Road Centre, South Eastern Sydney Local Health District, Sydney, NSW 1340, Australia" to "Centre for Population Health, Ministry of Health, Sydney, NSW 2065, Australia."

The original article has been corrected.
